# Supplementary figures and images for: A successful method to restore seagrass habitats in coastal areas affected by consecutive natural events
Source: PeerJ. 2024 Jan 2;12:e16700. doi: 10.7717/peerj.16700 (PMC10768669; doi:10.7717/peerj.16700)

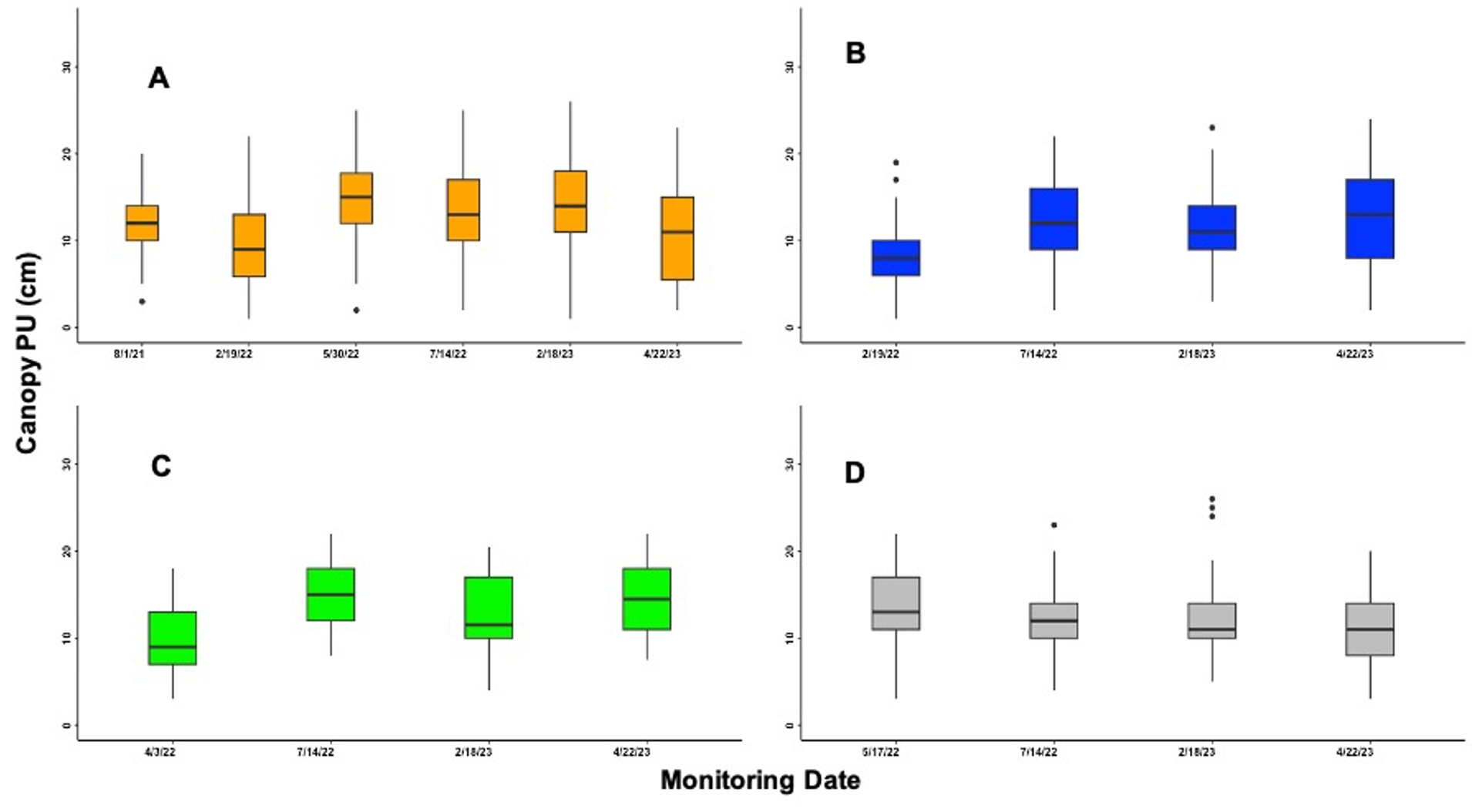

Supplement: Supplemental Information 2 — (A) The image showed Block A. (B) showed Block B. (C) Showed Block C. (D) Showed Block D. [file peerj-12-16700-s002.png]

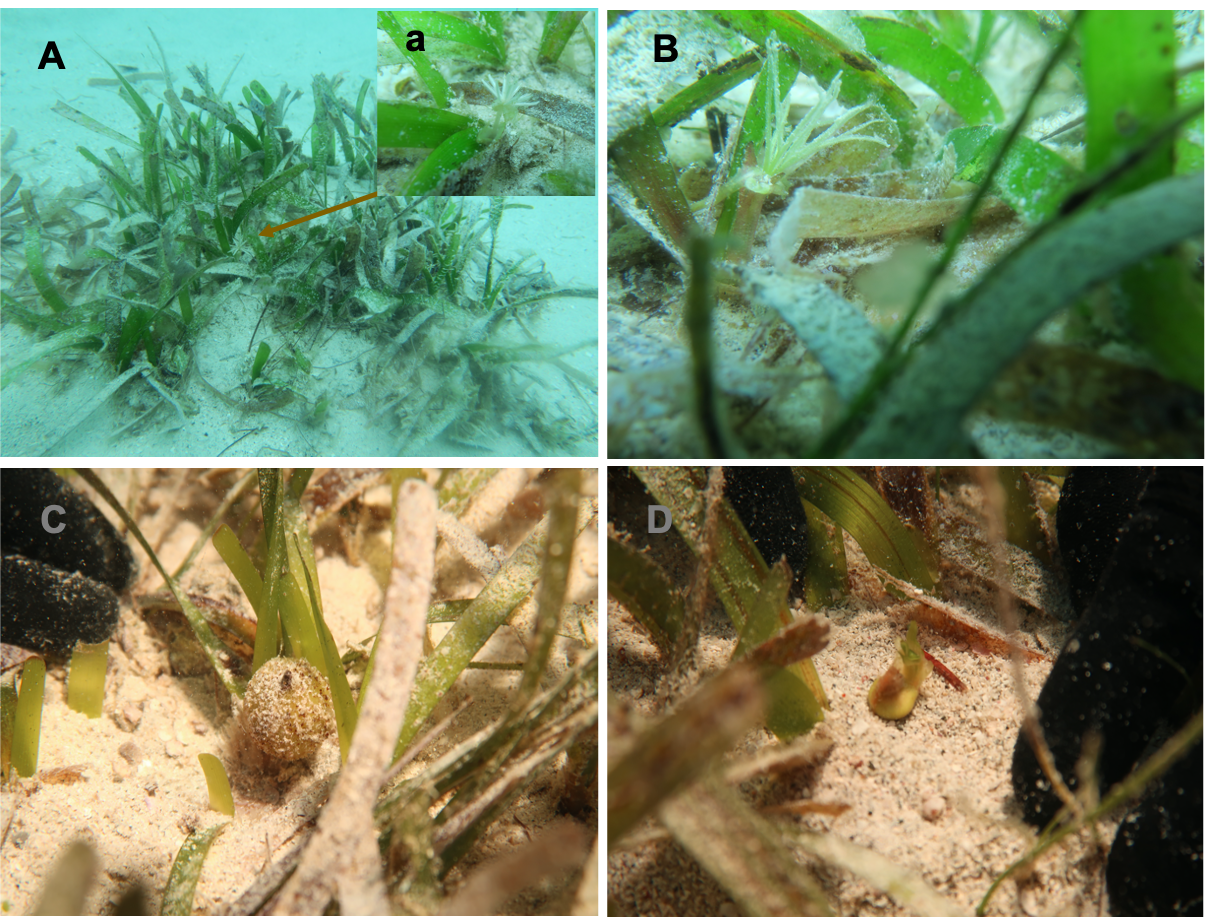

Supplement: Supplemental Information 3 — (A) male flower, (a) A close-up of the male flower. (B) The image shows the female flower of T. testudinum. (C) The image shows the fruit of T. testudinum. (D) A freshly germinated seed of T. testudinum. [file peerj-12-16700-s003.png]

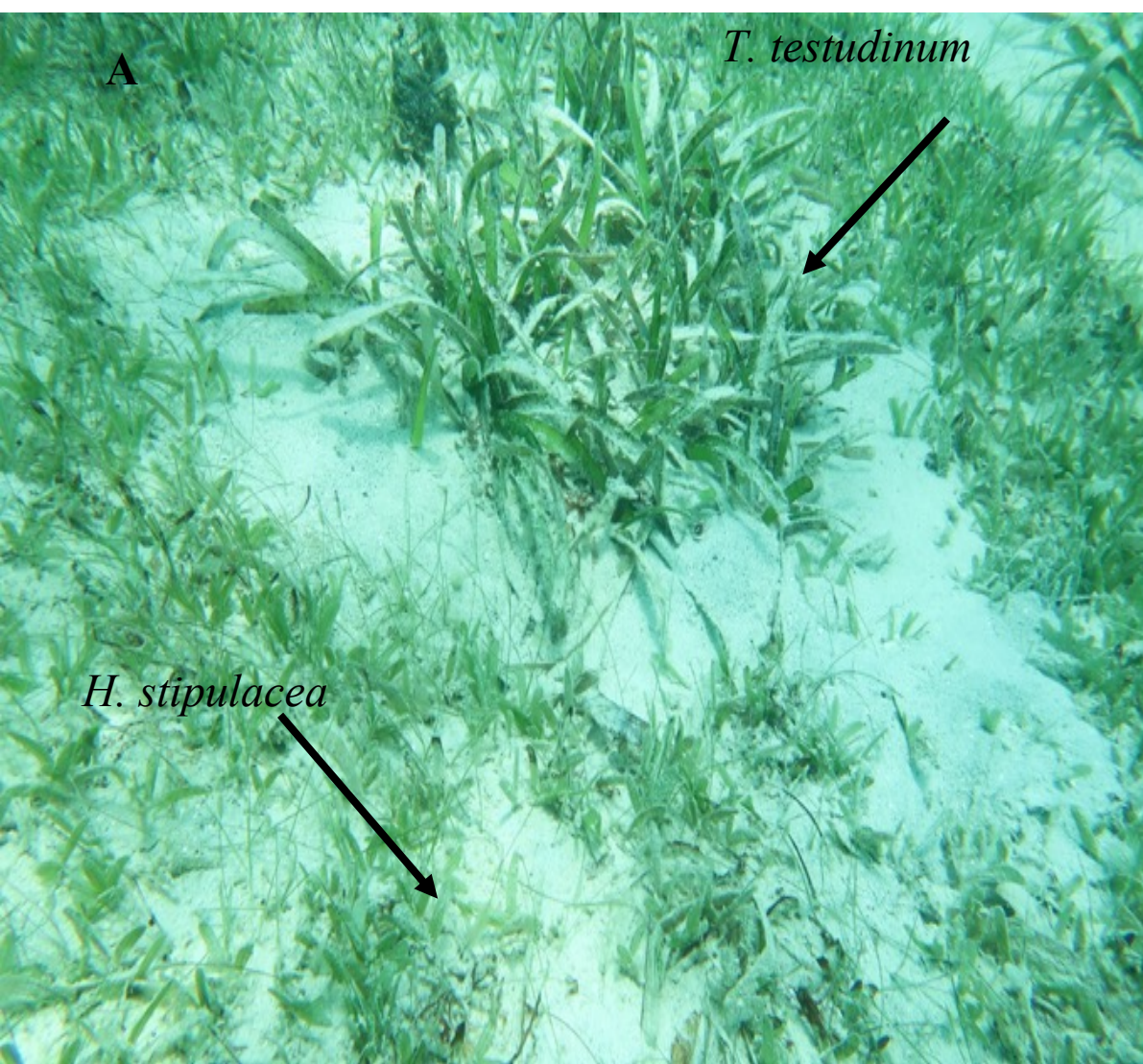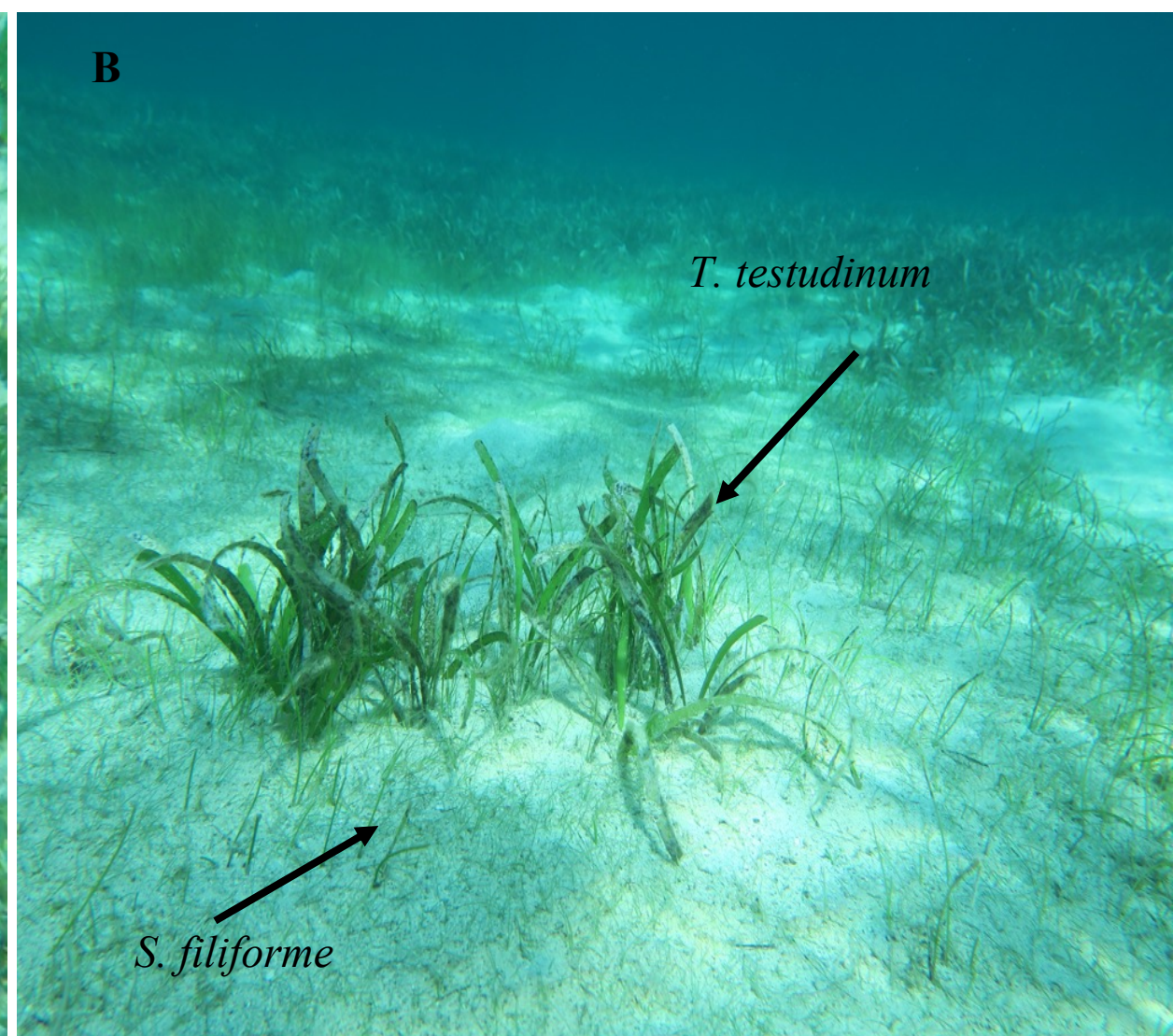

Supplement: Supplemental Information 4 — (A. PU was planted between the Halophila stipulacea area in May 2022. (B) Thalassia testudinum surrounded by Syringodium filiforme in April 2023. [file peerj-12-16700-s004.pdf]
